# The complete mitochondrial genome of the Great evening bat *Ia io* (Chiroptera: Vespertiilionidae) from karst area, Southwestern China

**DOI:** 10.1080/23802359.2022.2057247

**Published:** 2022-04-04

**Authors:** Peng-Fei Luo, Wei-Feng Wang, Xing-Liang Wang, Ya-Li Wang, Si-Wei Wang, Sha-Sha Yan, Qing-Qing He, Jiang Zhou

**Affiliations:** a School of Karst Science, Guizhou Normal University, Guiyang, China; b School of Life Sciences, Guizhou Normal University, Guiyang, China

**Keywords:** *Ia io*, mitochondrial genome, molecular tree, karst

## Abstract

In this study, the complete mitochondrial genome of *Ia io* from Guizhou Province, China. The genome was a circular mitochondrial genome of 16689 bp in length, containing 13 protein-coding genes (PCGs), 22 transfer RNA genes (tRNA), 2 ribosomal RNA genes (rRNA), and a control region. The average base composition is 32.76% A, 24.59% C, 14.49% G, and 28.16% T. The first complete mitochondrial genome of *I. io* provides fundamental data for future systematic taxonomic studies of the genus *Ia*.

The Great evening bat *Ia io* Thomas 1902, was not only the largest-sized bat among the family Vespertiilionidae, but also one of the few bird-eating and the egg-eating bats in existence (Thabah et al. 2007). Currently, *I. io* is widely distributed in southern China, including Guizhou, Chongqing, Sichuan, Yunnan, Guangdong, Guangxi, Jiangsu, Anhui, Hunan, Hubei, Jiangxi, Zhejiang, Jiangxi, and Hainan and South Asia, including Laos, Vietnam, Thailand, India, and Nepal (Bates and Harrison 1997; Bates et al. 2005; Jiang et al. 2021). Soisook et al. (2017) described the geographic populations of the Thai peninsula as a subspecies of *I. io*, namely *I. io peninsula*, based on morphological and molecular data. Some taxonomic problems still exist for the two subspecies of *I. io*, as Cyt b and COI sequences of specimens from Guizhou were not used. Therefore, a complete mitochondrial genome sequencing of specimens from China is necessary.

In this study, we collected the specimens of *I. io* from Guizhou Province (26°3′28.65″N, 105°7′48.73″E) and sequenced the complete mitochondrial genome using high-throughput sequencing (MZ579648). Currently, this specimen is kept at the Guizhou Normal University, Guiyang, Guizhou Province, China (voucher number: GZNU-NF-001; Jiang Zhou: zhoujiang@ioz.ac.cn). Total genomic DNA was extracted from the muscle tissue of the specimen using the CTAB method. The mitogenomes were sequenced by Illumina NovaSeq 6000 platform. Raw data (4.6 G) was deposited in the NCBI’s Sequence Read Archive database, and clean data (4.6 G) was subjected to *de novo* assembled by the SPAdes 3.14 (Bankevich et al. 2012) to produce a closed circular form of complete mitogenome.

The complete mitochondrial genome of *I. io* from Guizhou Province, which is 16,689 bp in length, with A + T contents of 60.92% (32.76% A, 28.16% T, 14.49% G, and 24.59% C), containing 13 protein-coding genes (PCGs), 22 transfer RNA genes (tRNA), 2 ribosomal RNA genes (rRNA), and a control region. Among the 37 genes, most of the genes were encoded on the H-strand, except for *tRNA^Gln^*, *tRNA^Ala^*, *tRNA^Asn^*, *tRNA^Cys^*, *tRNA^Tyr^*, *tRNA^Ser2^*, *tRNA^Glu^*, *tRNA^Pro^*, and *ND6*, which were encoded on the L-strand. Of the PCGs, two reading-frame overlap regions were observed between *ATP6* and *ATP8* genes (43 bp shared nucleotides), *ND4* and *ND4L* genes (1668 bp shared nucleotides). The start codon of most of the PCGs was ATG, except for *ND2* (ATT), *ND3* (ATA), and *ND5* (ATA). The stop codon of *Cyt b* was AGA, 7 PCGs (*ND1*, *ND4L*, *ND5*, *ND6*, *COI*, *COII*, *ATP6*, and *ATP8*) were TAA, while the rest were incomplete TA− (*ND3*) or T− (*ND2*, *ND4*, and *COIII*).

Combined with the mitochondrial genomes obtained in this study, we downloaded a total of 53 mitochondrial genomes from GenBank for phylogenetic analysis for Vespertilionidae, Molossidae, and outgroups. The 13 PCGs of the mitochondrial genomes were extracted in Phylosuite 1.2.2 (Zhang et al. 2020), and sequence alignment was performed using MAFFT (Katoh and Standley 2013). 13 PCGs were combined in Phylosuite 1.2.2 after the selection of the best-fit nucleotide substitution model was performed in PartitionFinder2 (Lanfear et al. 2017). Phylogenetic trees were reconstructed based on the best-fit model using the maximum likelihood method in IQ-TREE running 5000 ultrafast bootstrap (Nguyen et al. 2015). To combine the COI and Cyt b data from Soisook et al. (2017), we used Bayesian inference in MrBayes 3.2.1 (Ronquist et al., 2012) to assess the phylogenetic relationships of the two subspecies of the *I. io*.

Phylogenetic reconstructions using 13 mitochondrial protein-encoding genes showed that the genus *Ia* forms a highly supported evolutionary lineage within the Vespertiilionidae (Figure 1(A)), thus supporting the classification of *Ia* as an independent valid genus, as distinct from the taxonomic proposals of Tate (1942) and Simpson (1945). Reconstruction of the phylogenetic tree using Bayesian inference of the combined Cytb and COI showed that subspecies *I. io peninsulata* was mosaically distributed among three lineages comprising subspecies *I. io io* (Figure 1(B)), suggesting that the validity of subspecies *I. io peninsulata* needs further testing. In addition, the phylogenetic tree suggests that we need to focus on the skull and morphological differences between *I. io* from Yunnan, China, and Myanmar in the future.

**Figure 1.**
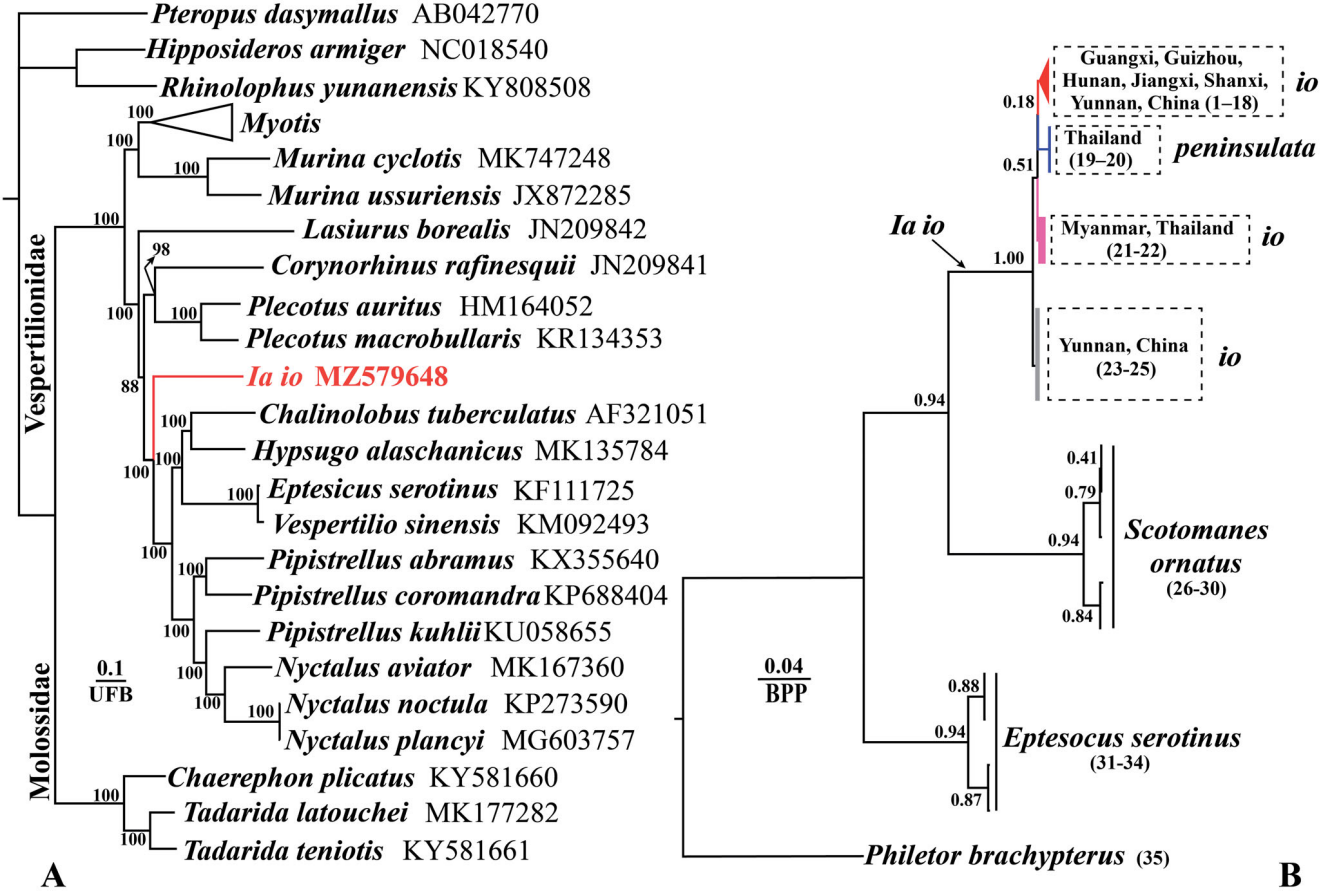
Phylogenetic tree reconstructed based on 13 protein-coding genes using maximum-likelihood (ML) (A) method and combined Cyt b and COI using Bayesian inference (BI) (B). ML tree and BI tree node numbers indicate the ultrafast bootstrap support value (UFB) and Bayesian posterior probability (BPP), respectively.

## Data Availability

The genome sequence data that support the findings of this study are openly available in GenBank of NCBI at (https://www.ncbi.nlm.nih.gov/) under the accession no. MZ579648. The associated BioProject, SRA, and Bio-Sample numbers are PRJNA748544, SRR15214614, and SAMN20336615 respectively.
